# Survey of prenatal screening policies in Europe for structural malformations and chromosome anomalies, and their impact on detection and termination rates for neural tube defects and Down's syndrome

**DOI:** 10.1111/j.1471-0528.2008.01700.x

**Published:** 2008-05-01

**Authors:** PA Boyd, C DeVigan, B Khoshnood, M Loane, E Garne, H Dolk

**Affiliations:** a National Perinatal Epidemiology Unit, University of Oxford Oxford, UK; b INSERM, UMR S149, Epidemiological Research Unit on Perinatal and Women's Health, Université Pierre et Marie Curie-Paris6 Paris, F75014, France; c Faculty of Life and Health Sciences, University of Ulster Newtonabbey, UK; d Department of Paediatrics, Kolding Hospital Kolding, Denmark

**Keywords:** Antenatal screening policy, Down's syndrome, neural tube defect, termination of pregnancy for fetal anomaly

## Abstract

**Objective:**

To ‘map’ the current (2004) state of prenatal screening in Europe.

**Design:**

(i) Survey of country policies and (ii) analysis of data from EUROCAT (European Surveillance of Congenital Anomalies) population-based congenital anomaly registers.

**Setting:**

Europe.

**Population:**

Survey of prenatal screening policies in 18 countries and 1.13 million births in 12 countries in 2002–04.

**Methods:**

(i) Questionnaire on national screening policies and termination of pregnancy for fetal anomaly (TOPFA) laws in 2004. (ii) Analysis of data on prenatal detection and termination for Down's syndrome and neural tube defects (NTDs) using the EUROCAT database.

**Main outcome measures:**

Existence of national prenatal screening policies, legal gestation limit for TOPFA, prenatal detection and termination rates for Down's syndrome and NTD.

**Results:**

Ten of the 18 countries had a national country-wide policy for Down's syndrome screening and 14/18 for structural anomaly scanning. Sixty-eight percent of Down's syndrome cases (range 0–95%) were detected prenatally, of which 88% resulted in termination of pregnancy. Eighty-eight percent (range 25–94%) of cases of NTD were prenatally detected, of which 88% resulted in termination. Countries with a first-trimester screening policy had the highest proportion of prenatally diagnosed Down's syndrome cases. Countries with no official national Down's syndrome screening or structural anomaly scan policy had the lowest proportion of prenatally diagnosed Down's syndrome and NTD cases. Six of the 18 countries had a legal gestational age limit for TOPFA, and in two countries, termination of pregnancy was illegal at any gestation.

**Conclusions:**

There are large differences in screening policies between countries in Europe. These, as well as organisational and cultural factors, are associated with wide country variation in prenatal detection rates for Down's syndrome and NTD.

*Please cite this paper as:* Boyd P, DeVigan C, Khoshnood B, Loane M, Garne E, Dolk H, and the EUROCAT working group. Survey of prenatal screening policies in Europe for structural malformations and chromosome anomalies, and their impact on detection and termination rates for neural tube defects and Down's syndrome. BJOG 2008;115:689–696.

## Introduction

Over the past 20 years, there have been major advances in the field of prenatal screening for Down's syndrome and in the efficacy of ultrasound scanning for the detection of fetal anomalies. Previously, older pregnant women were offered a diagnostic test (chorionic villus sampling [CVS] or amniocentesis, both associated with a risk for causing miscarriage) to detect Down's syndrome. Offering an amniocentesis to the oldest 5% of women identified about 30% of pregnancies with Down's syndrome.^1^ Today, a number of different noninvasive screening tests, which can be offered to women of any age, are available. These tests have different detection and false-positive rates.^2^–^4^

Improved resolution of ultrasound scans and greater expertise of operators have led to increased detection rates of fetal structural anomalies at earlier gestations. A variety of national policies or recommendations concerning prenatal screening and diagnostic testing for Down's syndrome and ultrasound screening for structural anomalies have been developed in different countries and areas within countries. One option for parents following prenatal diagnosis of fetal anomaly is termination of pregnancy. Termination of pregnancy is a controversial subject in many countries, and the laws governing it and legal gestation limits vary.

This study aims to ‘map’ the current (2004) state of prenatal screening and diagnosis in 18 countries in Europe that are members of EUROCAT and to relate them to prenatal detection and termination of pregnancy rates for specific anomalies.

## Methods

EUROCAT (European Surveillance of Congenital Anomalies) is a network of population-based congenital anomaly registers in Europe.^5^ Full member registries of EUROCAT send case data on all congenital anomalies in their region, while associate member registries send aggregate data only based on pregnancy outcome and congenital anomaly subgroup. One of the objectives of EUROCAT (which surveys more than 1.5 million births per year, including information on termination of pregnancy for fetal anomaly [TOPFA] and covers 29% of the annual European birth population) is to assess the impact of prenatal screening.

A questionnaire was developed to explore current national policies or recommendations in place in the year 2004 concerning prenatal screening for congenital anomalies (Down's syndrome and structural anomalies) and the laws relating to TOPFA.^6^ As well as determining the existence, or not, of an official country-wide policy or recommendation, the questionnaire covered the tests actually offered throughout each country. The questionnaire was filled in by a EUROCAT register leader (clinician or public health professional) from each participating country. Information about each participating register is available on the EUROCAT website.^5^

Data on Down's syndrome and neural tube defects (NTDs) not associated with an abnormal karyotype were analysed. Full EUROCAT member registries with information on gestation at diagnosis known for at least 80% of cases, date of delivery between 2002 and 2004 and data on termination of pregnancy as well as on births were included in the data analysis. Multiple pregnancies were excluded. Data on total number of cases, prenatal detection and termination of pregnancy rates with median gestational age at prenatal diagnosis were extracted from the EUROCAT central database.^5^ We calculated the percentage of cases prenatally diagnosed and the percentage of pregnancies resulting in termination with 95% binomial exact confidence intervals.

For assessing the relation between the proportion of cases with a prenatal diagnosis and country policies, we used risk differences as the measure of association.^7^ For each policy category, the risk difference represented the difference between the overall proportion of cases with a prenatal diagnosis for countries with that policy and the overall proportion of cases with a prenatal diagnosis for countries with the policy category of reference. The same methodology was used to assess risk differences in TOPFA.

For Down's syndrome, the policy category of reference for prenatal screening was first or second-trimester screening for the whole country; for NTD, the policy category of reference was having a national policy in place for routine ultrasound. For both malformations, the policy category of reference for pregnancy termination was no legal gestational age limit. Reference categories were chosen so as to represent the most frequent policy category. We used binomial regression,^8^ weighted by the number of cases for each country, to estimate the risk differences with exact 95% CIs. Risk differences were considered statistically significant if the 95% CI did not include zero.

## Results

Eighteen questionnaires covering 18 countries were completed. The countries, their EUROCAT registers, total number of births in each country, and the number of births in areas within the countries covered by the EUROCAT registers in 2002 are shown in Table 1.

**Table 1:** Eighteen countries with EUROCAT membership, total number of births in 2002, number and percentage of births covered by EUROCAT registries

Countries	EUROCAT register(s)	Total births in country in 2002**	Number (%) of births in areas covered by EUROCAT register(s)***
Austria	Styria	72 900	10 500 (14)
Belgium	Antwerp, Hainaut	113 300	29 500 (26)
Croatia	Zagreb	43 000	5500 (13)
Denmark	Odense	64 800	5100 (8)
England and Wales	NorCAS, North West Thames, Oxford, Trent, Wessex, Wales	662 200	192 800 (29)
Finland	Finland*	55 800***	55 800 (100)
France	Auvergne, Paris, Central East*, Strasbourg	773 500	159 400 (21)
Germany	Mainz, Saxony-Anhalt	741 600	20 900 (3)
Ireland	Cork and Kerry, Dublin	60 500***	31 235 (52)
Italy	Campania, Emilia Romagna, Northeast Italy, Tuscany	522 900	176 800 (34)
Malta	Malta	3800***	3800 (100)
Netherlands	North Netherlands	209 300	20 400 (10)
Norway	Medical Birth registry of Norway*	56 500***	56 500 (100)
Portugal	Southern Portugal	124 800	19 000 (15)
Poland	Wielkopolska, Poland*	386 000	253 300 (66)
Spain	Barcelona, Basque, Asturias, Madrid*	413 000	148 900 (36)
Sweden	Sweden*	96 200***	96 200 (100)
Switzerland	Vaud	73 000	6800 (9)

* Associate member of EUROCAT.

** 2002 World Population Data Sheet, Population Reference Bureau www.prb.org/pdf/WorldPopulationDS02_Eng.pdf.

*** EUROCAT website: www.EUROCAT.ulster.ac.uk.

Table 2 shows the legal gestational age limit (if any) for TOPFA in different countries.

**Table 2:** National laws regarding TOPFA laws categorised by legal gestational age limit

No legal gestational age limit	No legal gestational age limit if lethal	Legal gestational age limit ≤28 weeks	Not legal at any gestation
Austria	Netherlands	Finland	Ireland
Belgium	Norway	Italy	Malta
Croatia	Portugal	Poland*	
England and Wales	Denmark	Spain	
France		Sweden	
Germany		Switzerland	

* Only for severe malformations.

### Down's syndrome

Table 3 outlines which countries had national screening policies/recommendations for Down's syndrome in place in 2004, the maternal age ‘cutoff’ (if any) at which diagnostic testing by CVS or amniocentesis is usually offered and the type of screening offered (first trimester—nuchal scan alone or combined with biochemistry or second-trimester biochemical screening).

**Table 3:** National policies or recommendations for prenatal screening for Down's syndrome in place in 2004 in 18 European countries

		First-trimester screening actually offered			
Countries	National screening policies or recommendations for Down's syndrome screening test to be offered to all women	Nuchal scan	Nuchal + biochemistry	Second-trimester biochemical screening offered	Maternal age at which CVS/amniocentesis are offered
Austria	No	+	+	−	≥35
Belgium	Yes	+		+	≥36 (charged if <36)
Croatia	No	±	±	±	≥35
Denmark	Yes	−	+	−	CVS/amniocentesis not offered primarily on basis of maternal age
England and Wales	Yes*	±	±	±	CVS/amniocentesis not offered primarily on basis of maternal age
Finland	Yes	±	±	±	≥39
France	Yes	+	±	+	≥38
Germany	Yes	+**	+**	+**	≥35
Ireland	No	−	−	−	—
Italy	Yes	±	±	+	≥35
Malta	No	−	−	−	—
Netherlands	No	−	−	+**	≥36
Norway	No	±**	±**	±**	≥38
Poland	Yes	+	+	+	≥35
Portugal	Yes	+	±	±	≥35
Spain	No	±	±**	±**	≥35
Sweden	No	±	−	−	≥35
Switzerland	Yes	−	+	±***	CVS/amniocentesis not offered primarily on basis of maternal age

+, in place in all areas of country; ±, in place in some areas within country.

* Screening policy was based on a detection rate, that is a screening test should be offered that had a detection rate for Down's syndrome of >60% for a false-positive rate of <5%.

** May be private.

*** Primarily first-trimester screening, second-trimester screening for late bookers.

Table 4 shows the total number of cases of Down's syndrome (from the 12 full EUROCAT registries meeting the inclusion criteria), percentage prenatally diagnosed, median gestation at diagnosis and the number and percentage resulting in termination of pregnancy.

**Table 4:** Number of Down's syndrome cases delivered in 2002–04, percentage prenatally diagnosed, median (range) weeks of gestation at prenatal diagnosis and number and percentage resulting in termination of pregnancy in 19 EUROCAT registry areas in 12 countries

			Prenatal diagnosis			Termination of pregnancy		
Countries	Screening policies*	Total cases of Down's syndrome	Number of cases	% (95% CI)**	Median gestation (weeks) at detection (range)	Number of cases	% of prenatally diagnosed cases (95% CI)**	% of total cases (95% CI)**
Denmark	A	22	14	64 (41–83)	11 (10–30)	12	86 (57–98)	55 (32–76)
Switzerland	A	60	57	95 (86–99)	15 (10–35)	52	91 (81–97)	87 (75–94)
Belgium	B	79	53	67 (56–77)	19 (12–25)	48	91 (79–97)	61 (49–72)
England and Wales	B	652	429	66 (62–70)	17 (10–40)	325	76 (71–80)	50 (46–54)
France	B	455	408	90 (87–92)	16 (11–35)	392	96 (94–98)	86 (83–89)
Germany	B	36	23	63 (46–79)	15 (12–36)	22	96 (78–100)	61 (44–77)
Italy	B	536	380	71 (67–75)	19 (10–40)	352	93 (90–95)	66 (62–70)
Croatia	C	22	7	32 (14–55)	17 (17–17)	7	100 (59–100)***	32 (14–55)
Netherlands	C	88	37	42 (32–53)	14 (10–35)	27	73 (56–86)	31 (21–41)
Spain	C	204	153	75 (68–81)	16 (11–29)	147	96 (92–99)	72 (65–78)
Ireland	D	130	7	5 (2–11)	26 (13–35)	0	0	0
Malta	D	24	0	—	0	0	—	—
Total		2308	1568	68 (66–70)	17 (10–40)	1384	88 (87–90)	60 (58–62)

* A, first-trimester screening offered in whole country; B, first- or second-trimester screening offered in whole country; C, no national policy but some form of screening in some of country; D, no screening.

** 95% binomial exact confidence intervals.

*** One-sided 97.5 CI.

Of the 2308 cases of Down's syndrome, 68% (95% CI 66–70) were diagnosed prenatally at a median gestation of 17 weeks (range 10–40 weeks) and 1384 (60%, 95% CI 58–62) of all affected pregnancies resulted in termination of pregnancy. If Malta and Ireland (where TOPFA is illegal) are excluded from the analysis, of the 2154 cases of Down's syndrome, 73% (95% CI 71–74) of cases were prenatally diagnosed at a median gestation of 16 weeks (range 10–40 weeks) and 64% (95% CI 62–66) of all affected pregnancies resulted in TOPFA.

Those two countries (Denmark and Switzerland) with a primarily first-trimester screening policy had a proportion of prenatally diagnosed cases 13% (95% CI 5–21) higher than those countries with first- or second-trimester screening, the reference group; those with no policy but some screening (three countries: Croatia, Netherlands and Spain) had a proportion of prenatally diagnosed cases 11% (95% CI 5–17) lower than the reference group.

A Down's syndrome case was 28% (95% CI 19–38) less likely to result in termination of pregnancy in those countries with a legal limit for nonlethal anomalies than those with no legal gestational age limit for termination (the reference group). Down's syndrome cases from countries with a legal gestation limit for termination of less than or equal to 28 weeks were 5% (95% CI 1–9) more likely to result in termination compared with the reference group.

### Ultrasound screening and detection of NTDs

Table 5 shows which countries had a national policy or recommendation in place in 2004 for routine prenatal ultrasound scanning and the number and gestation at which the scans are performed.

**Table 5:** National policy/recommendations for routine prenatal ultrasound scans in place in 2004 in 18 European countries

Countries	Routine ultrasound scan policy/recommendations	Gestation at routine scans (weeks)
Austria	Two scans	10–14*, 18–22, 30–34
Belgium	Three scans	10–14, 18–23, 29–33
Croatia	One scan	10–14*, 18–23, 34–37*
Denmark	Two scans	10–14 (nuchal), 18
England and Wales	Two scans	10–12, 18–23
Finland	One or two scans	16–19 if only one scan, 13–14 and 18–20 if two scans
France	Three scans	10–14, 18–23, 29–32
Germany	Three scans	9–12, 19–22, 29–32
Ireland	No national policy	18–22*
Italy	Three scans	10–14, 18–23, 30
Malta	No national policy	18–23*, 34–25*
Netherlands	No national policy	No routine scans
Norway	One scan	18
Portugal	Three scans	10–14, 18–23, 29–33
Poland	Three scans	11–14,18–22, 28–32
Spain	No national policy—practice varies between regions	10–14*, 18–23*, 29–33*
Sweden	Two scans	10–14, 16–17
Switzerland	Two scans	11–14, 20–22

* Not official policy but usually performed.

Fourteen of the 18 (78%) countries had a national policy or recommendation regarding fetal ultrasound scanning in place in 2004. These were for a specific anomaly scan at 18–23 weeks (Sweden 16–17 weeks, Finland 16–19 weeks) with, in most countries, additional scans at 10–14 and 28–32 weeks. There were no national scan policies in place in Ireland, Malta or Spain, but anomaly scans were routinely offered. Routine scans were not offered in the Netherlands.

Table 6 shows the total number and percentage of cases of NTD (from full EUROCAT registry areas in each of the 12 countries providing data), percentage prenatally diagnosed, median gestation at diagnosis and the number and percentage resulting in termination of pregnancy. Of the 725 NTD cases, 88% (95% CI 86–90) were detected prenatally at a median gestation of 17 weeks (range 8–40 weeks). Five hundred and sixty out of 725 (77%, 95% CI 74–80) of all affected cases were electively terminated. If Malta and Ireland (where TOPFA is illegal) are excluded from the analysis, of the 669 NTD cases, 91% (95% CI 88–93) were prenatally diagnosed at a median gestation of 17 weeks (range 8–40 weeks) and 84% (95% CI 81–86) of all affected pregnancies resulted in TOPFA.

**Table 6:** Total number of cases of NTDs, percentage prenatally diagnosed, median (range) weeks of gestation at prenatal diagnosis and number and percentage resulting in termination of pregnancy for 12 countries with EUROCAT registries

			Prenatal diagnosis			Termination of pregnancy		
Countries	Ultrasound policies*	Total cases of NTD	Number of cases	% of total cases (95% CI)**	Median gestation (weeks) at detection (range)	Number of cases	% of prenatally diagnosed cases (95% CI)**	% of total cases (95% CI)**
Belgium	1	23	19	83 (61–95)	16 (11–29)	17	89 (67–99)	74 (52–90)
Croatia	1	5	4	80 (28–100)	12 (8–16)	4	100 (40–100)***	80 (28–100)***
Denmark	1	9	8	89 (52–100)	16 (12–36)	7	88 (47–100)	78 (40–97)
England and Wales	1	281	264	94 (91–96)	17 (10–40)	242	92 (88–95)	86 (82–90)
France	1	109	102	94 (87–97)	14 (10–32)	100	98 (93–100)	92 (85–96)
Germany	1	10	9	90 (56–100)	18 (11–34)	4	44 (14–79)	40 (12–74)
Italy	1	137	119	87 (80–92)	18 (10–39)	112	94 (88–98)	82 (74–88)
Switzerland	1	12	10	83 (52–98)	13 (12–18)	10	100 (69–100)***	83 (52–98)
Ireland	2	48	27	56 (41–71)	22 (16–39)	0	0 (0–13)***	0
Malta	2	8	2	25 (3–65)	19 (19)	0	0 (0–84)***	0
Spain	2	65	61	94 (85–98)	16 (11–22)	60	98 (91–100)	92 (83–98)
Netherlands	3	18	14	78 (52–94)	31 (16–40)	4	29 (8–58)	22 (6–48)
Total 12		725	639	88 (86–90)	17 (8–40)	560	88 (85–90)	77 (74–80)

* 1, national ultrasound scan policy; 2, no national scan policy but routine scans carried out; 3, no routine scans.

** 95% binomial exact confidence intervals.

*** One-sided 97.5 confidence limit.

Those three countries (Ireland, Malta and Spain) with no national ultrasound policy in place but where routine scans were carried out had a proportion of prenatally diagnosed NTD cases 17% (95% CI 9–25) lower than those countries with a country-wide policy, the reference group. The one country (Netherlands) with no policy and no routine scans carried out had a proportion of prenatally diagnosed cases 14% (95% CI 6–33) lower than the reference group.

A NTD case was 45% (95% CI 26–64) less likely to result in termination of pregnancy in those countries with a legal gestation limit for nonlethal anomalies than those with no legal gestational age limit for termination (the reference group). NTD cases from countries with a legal gestation limit for termination of less than or equal 28 weeks were 1% (95% CI −7 to +5) more likely to result in termination compared with the reference group.

## Discussion

This attempt to ‘map’ the state of prenatal diagnosis in 18 European countries in 2004 has confirmed wide variation in the availability and type of noninvasive screening tests for Down's syndrome, in the number of ultrasound scans offered and in the legal gestational limits regarding TOPFA. This broad view of prenatal diagnosis updates a previous report describing prenatal diagnosis in different countries in Europe between 1993 and 1995.^9^

In 2004, the majority of countries had moved from solely offering older mothers a diagnostic test to having some form of Down's syndrome screening in place, with over half having an official country-wide policy or recommendation for first- or second-trimester screening. Having a screening policy in place had a measurable impact on prenatal detection rates for Down's syndrome; the registry areas in countries offering primarily first-trimester screening had a significantly higher detection rate than those using a mixed first or second-trimester screening policy; those with some screening but no national screening policy in place were significantly less likely to detect a Down's syndrome case prenatally. However, there are wide variations in detection rates between different countries using similar screening policies. For example, Germany and France have both first and second-trimester screening policies; yet, the prenatal detection rate of Down's syndrome in the German registry area is 63% compared with 90% for France. Some of this difference may be due to a higher proportion of older mothers in the French registry (Paris, 28% of mothers aged ≥35 years) than in the German registry area (Mainz, 22% of mothers aged ≥35 years) in the period 2002–04. In all countries where TOPFA is legal, the majority of cases of Down's syndrome detected prenatally resulted in termination of pregnancy; in most (7/10) registry areas, more than 90% of prenatally diagnosed affected pregnancies resulted in termination.

Most countries had an official, country-wide policy for routine ultrasound anomaly scanning. This study has used the prenatal detection of NTDs as an indicator for assessing the efficacy of ultrasound anomaly scanning because they are relatively common and are associated with a high prenatal detection rate.^10^ It may, however, be that other anomalies which are more difficult to detect prenatally would serve as better indicators of the widespread use and quality of ultrasound anomaly screening. Those countries that did not have a policy for offering routine scans had a significantly lower prenatal detection rate of NTD. One factor that may be important when termination of pregnancy is being considered is the gestational age at suspicion of fetal anomaly. Of the countries where TOPFA is legal, the country with the lowest termination rate for NTD (29% of prenatally detected cases) was the Netherlands where the median gestation at prenatal diagnosis was 31 weeks compared with 17 weeks for all countries. However, gestation at diagnosis is not the only factor; in the German registry area, 90% of NTDs were prenatally detected at a median gestation of 18 weeks and less than half (44%) of the prenatally detected cases resulted in termination of pregnancy, while in France, England and Wales and Spain, there were high detection rates (94%) and high (92–98%) termination rates.

Discussion of TOPFA and the legal gestation limit for termination causes controversy. The laws regarding TOPFA vary in their gestation limit in the different countries. This study shows that having a legal limit of less than 28 weeks of gestation for TOPFA does not have a major impact on termination rates for Down's syndrome or NTDs. This implies that a well-organised screening system should be able to ensure that most women are given choices before the fetus becomes viable.

A strength of this study is that it provides information on prenatal policies in place in whole countries rather than in centres of excellence and attempts to relate policies or lack of policies to prenatal detection rates. However, there are limitations; first, because data are only provided from areas covered by full EUROCAT registries; for some countries (e.g. Switzerland and Germany), this will only be from a small area, which is not necessarily representative of the whole country. Second, although the year 2004 has been chosen for the existence or not of a country-wide policy, data are from the years 2002–04. For one country (Denmark, where the screening policy was introduced in 2004), this may underestimate the impact of a recently introduced policy.

The existence of a national policy or recommendation for a particular screening test does not necessarily equate with the delivery of the offer of such screening to all women in all areas because of lack of resources, of information provided to women within the time frame for making an informed decision, lack of uptake or late booking.^11^,^12^ The uptake and impact of different programmes will depend on social and cultural factors as well as on the availability of different resources and laws regarding TOPFA. The absence of a national screening policy may reflect a considered decision that is itself ‘a policy’.

In this paper, we have concentrated on the two anomalies (Down's syndrome and NTDs) for which screening methods were initially developed. The existence of screening has led to difficult ‘grey areas’ in terms of what types of birth defect can now be prenatally detected and whether termination of pregnancy is an appropriate choice, for example Turner syndrome and facial clefts.^13^–^15^ There is some concern about a potentially negative effect of widespread screening on the perceptions about individuals born with birth defects and the services that might be available for their care.^13^,^14^ However, prenatal screening has opened up new possibilities to enhance the treatment and survival of liveborn children with birth defects.^16^,^17^

The situation regarding screening policies is not a static one. Some countries will have already updated their policies to achieve higher detection and lower false-positive rates. New developments in noninvasive prenatal testing based on fetal DNA in maternal blood are becoming a realistic prospect for the future.^18^,^19^ We can expect that prenatal screening policy will continue to be dynamic and that variation between countries in Europe will continue to lead to large but changing differences in prenatal detection and termination rates.

## Conclusion

Prenatal screening policies as well as prenatal diagnosis and TOPFA rates for Down's syndrome and NTDs vary widely across European countries. The majority of Down's syndrome and NTD cases are prenatally detected. Having a legal gestational age limit for TOPFA does not significantly alter the number of pregnancies resulting in TOPFA. National policy is associated with prenatal detection rate, but organisational and cultural factors are clearly important.

## Funding

EUROCAT is funded by the European Commission Public Health Programme. P.A.B. is funded at the National Perinatal Epidemiology Unit by the Department of Health in England. The views expressed in this paper are those of the authors and do not necessarily reflect the views of the Department of Health.

## Contribution to authorship

E.G., C.D.V., P.A.B. and H.D. designed the study, drafted the questionnaire and designed the data analysis. P.A.B. led the writing of the paper and coordinated the study. M.L. coordinated and analysed the data, B.K. carried out the statistical analysis. All authors contributed to writing and editing the paper.

## Ethical approval

All registries have ethical approval appropriate to their national and local ethics guidelines.
